# Intercepting accelerated moving targets: effects of practice on movement performance

**DOI:** 10.1007/s00221-017-4895-6

**Published:** 2017-02-14

**Authors:** João V. A. P. Fialho, James R. Tresilian

**Affiliations:** 1 Grupo de Estudos em Ciências do Movimento e da Saúde, Curso de Educação Física, Centro Universitário CESMAC, Rua Conêgo Machado, 918-Farol, Maceió-Al, 57051-160 Brazil; 2 Department of Psychology, The University of Warwick, Coventry, UK

**Keywords:** Human, Interception, Acceleration, Practice, Movement, Timing

## Abstract

When performing a rapid manual interception, targets moving under constant motion are often intercepted with greater accuracy when compared to targets moving under accelerated motion. Usually, accelerated targets are timed too late and decelerating ones too early. The present experiment sought to investigate whether these differences in performance when intercepting targets moving under constant and accelerated motions change after a short period of practice. The task involved striking targets that moved along a straight track by moving forward a manipulandum that moved along a slide perpendicular to the target’s motion. Participants were allocated to one of the three experimental groups, defined according to the type of motion of the moving targets: constant speed, constant acceleration, and constant deceleration. Results showed that after some practice participants were able to intercept (positive and negative) accelerating moving targets as accurately as constant speed targets. These results suggest that people might be able to learn how to intercept accelerating targets, corroborating the results of some recent studies.

## Introduction

People’s responses to moving objects are of two basic types: responses in which the object is contacted—called interceptive responses or interceptive actions (Zago et al. 2009, for a recent review)—and those in which contact is avoided. These responses involve changing the position of the body or a body part, so that it moves into or out of the moving object’s path (positioning) and doing so at the right time (timing; Lee 1980). Control of response timing has been studied in greater detail than the control of positioning. One reason for this is that response timing can be studied independently of positioning using the coincidence anticipation task in which a person attempts to make a discrete response, such as a button press, at the same moment that a moving object arrives at a specified location (Payne 1986; Schmidt and Lee 2011). Another reason is that a controversial hypothesis concerning the perceptual basis for timing control—the tau-hypothesis (Lee 1980)—has been the subject of many empirical studies (see Tresilian 1999; Wann 1996).

One of the claims of the tau-hypothesis is that the acceleration (specifically, the rate of change of speed) of a moving object is not taken into account in the timing of interceptive actions (Lee and Reddish 1981). The finding that the human visual system is very poor at detecting and estimating the rate of change of speed (Watamaniuk and Heinen 2003; Werkhoven et al. 1992) lends some support to this idea; a number of empirical studies of human timing performance are also consistent with it when accelerations of both gravitational magnitude (= 9.81 m/s^2^) (Lee et al. 1983; Michaels et al. 2001) and non-gravitational magnitude (Benguigui et al. 2003; Port et al. 1997; Senot et al. 2003) were involved.

Although it was clear early on that there were a few interceptions that would be impossible to achieve if acceleration were not being taken into account, specifically those involving short falls (<2 m) from rest under gravity (Lacquaniti and Maioli 1989; Tresilian 1993), it is possible that these are special cases that people learn to deal with in a different way (e.g., using the drop height to determine the timing, Tresilian 1993; Wann 1996). However, subsequent studies of interceptions under gravitational magnitude acceleration over greater distances demonstrated that people were able to use internalised information about the gravitational acceleration (a kind of internal model) to time their responses (McIntyre et al. 2001; Zago et al. 2004, 2005). It is possible that knowledge of the gravitational acceleration is acquired over years of experience with falling objects and so other accelerations might be treated differently. This is plausible given that the magnitude of gravitational acceleration is large compared with other, naturally occurring, continuous accelerations: ignoring the latter would lead to much smaller errors than ignoring gravitational acceleration and Lee’s argument that accelerations can be ignored without incurring large errors could plausibly apply (Lee and Reddish 1981; Lee et al. 1983). However, recent evidence indicates that is not always the case: relevant knowledge of accelerations having gravitational and smaller, non-gravitational magnitudes can be acquired relatively quickly and used in the control of timing (de Rugy et al. 2012; La Scaleia et al. 2014; Tresilian and Lonergan 2002; Zago et al. 2004).

De Rugy et al. (2012) employed a coincidence anticipation task (participants pressed on a force sensor) in which the moving object underwent an accelerative perturbation. The object was a simulated ball rolling down a tube at constant speed; shortly before arriving at the specified target location the tube curved into one of several different concave (downwards) or convex (upwards) humps so that the ball accelerated and decelerated. This affected the time it took to reach the target line. Results showed that participants were able to compensate for most (about 85%) of the effect of these perturbations on the ball’s time to arrival within about 300 trials (de Rugy et al. 2012). Performance in constant speed catch trials demonstrated that compensation was (at least in large part) anticipatory rather than an acquired feedback-based reaction to specific stimulus conditions. Although this finding does not demonstrate the acquisition of an internal model of the magnitude of the acceleration, it does demonstrate a role for acquired knowledge in controlling the timing of interceptions involved accelerations of a non-gravitational magnitude.

Less clear is how people deal with continuous, constant accelerations of non-gravitational magnitudes such as those produced when a moving object rolls or slides up or down an inclined plane or over a flat, frictional surface (La Scaleia et al. 2014). It is known that human observers exhibit a number of cognitive misunderstandings about accelerated motions up and down slopes (e.g., Bertamini 1993; Hecht 1993; Rohrer 2002), including neglect of acceleration (Ebersbach et al. 2011; studies by Bozzi described in; La Scaleia et al. 2014). This suggests that people might show systematic timing errors when intercepting objects undergoing these motions (La Scaleia et al. 2014), which is consistent with the previous studies using accelerations of non-gravitational magnitude (Benguigui et al. 2003; Port et al. 1997). However, as La Scaleia et al. (2014) note, there has been surprisingly little empirical study of actually intercepting objects rolling up or down slopes. In one relatively early study, people were found to be able to consistently achieve a high degree of temporal accuracy (to within ±17 ms) within relatively few practice trials (typically <50) when hitting targets accelerating under gravity down an inclined track at 1.2 or 2 m/s^2^ (Tresilian and Lonergan 2002, though these authors did not report on how performance changed with practice). Similar findings were reported by La Scaleia et al. (2014) over a much wider range of conditions and using both interceptive and non-interceptive responses. In both these studies, the targets accelerated down an incline (so speed increased) and the incline itself was visible throughout, as was the target’s starting location. Thus, neither study examined interception of decelerating targets. Participants in both studies could also view the incline before and during the interception and could see the starting location of the target, which provided advance cues about the target’s motion that participants could have learned to use to predict a target’s time to arrival.

In the study reported here, we examined whether people could learn to accurately time interceptions of a moving target that accelerated or decelerated at a constant rate (1.2 m/s^2^) and we report how their performance changed with practice. The target’s motion was constrained to a horizontally oriented track, so there were no cues provided by an incline. In addition, the participants were provided with no information about the initial conditions of motion (starting position or speed) that could be used to predict time to arrival. The results add to our understanding of the range of conditions in which people can learn to take accelerations into account when timing interceptive actions.

## Method

The experimental procedures described in this manuscript were approved by the Ethics Committee of The University of Warwick and were in accordance with the ethical standards of the national research committee and with the 1964 Helsinki declarations and its later amendments or comparable ethical standards.

### Participants

Twenty-one psychology students (21 men, age range: 19–25 years) from The University of Warwick participated voluntarily in the experiments and gave their informed consent prior to commencement of the testing sessions. They all received course credits for their participation. Seven students were pseudorandomly assigned to each experimental group. All were self-reported right handed and had normal or corrected to normal vision.

### Apparatus and task

Participants were required to perform a task which consisted of striking a moving object with an intercepting effector (manipulandum), in a plane perpendicular to the target’s motion, performing a brief hitting movement. Participants were constrained to move the manipulandum along one single spatial dimension performing a movement characterized as a one degree of freedom (1*df*).

**Fig. 1:**
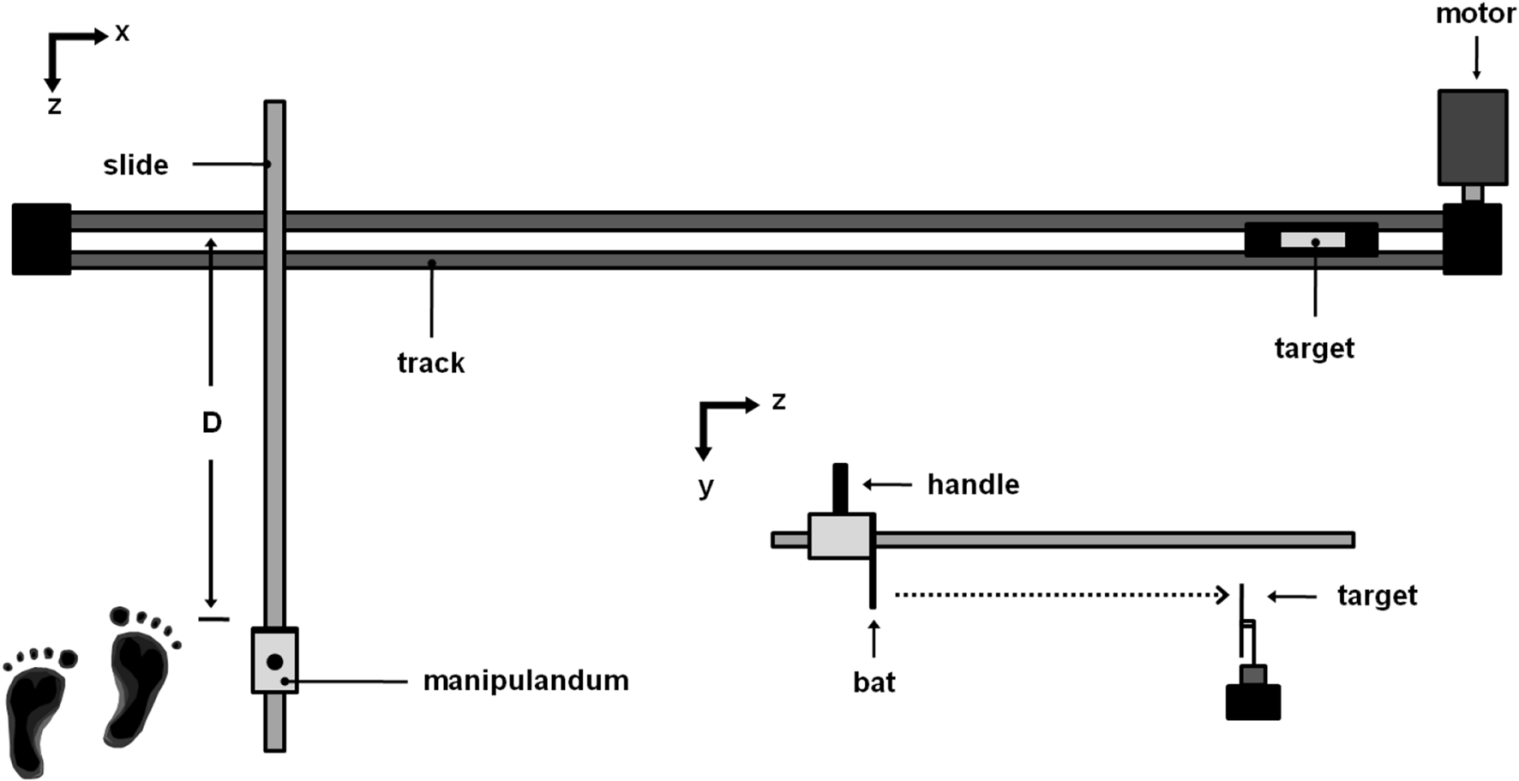
Schematic diagram of the hitting task showing the setup used in the experiment. The target is attached to the track belt and moves along a straight path. The intersection between the target track and the manipulandum track determines the position in which the target must be intercepted. The participants were constrained to move the manipulandum only along the *Z* axis. Participant’s position is indicated by the schematic feet

A schematic diagram of the experimental apparatus is shown in Fig. 1 and was similar to the one used in the previous experiments (Tresilian et al. 2003). The target was fixed on a 2.5 cm wide belt, which was driven by a computer controlled torque motor (Baldor Motors & Drivers) around two pulleys 5 m apart. Participants moved a hand-held manipulandum along a linear slide (Starr Industries, Germany) 9.5 cm above and perpendicular to the target drive belt. The manipulandum consisted of a plastic handle mounted on a rectangular plastic block housing a bearing that runs with minimal friction along the linear slide. The target was struck by an aluminium rod (bat), 0.5 cm diameter, which was attached to the front end of the plastic block. To strike the target, the manipulandum had to be moved through a distance (*D*) of 18.4 cm.

The target was flat and rectangular, 5 cm tall and 7.5 cm in length, made of aluminium material covered with bright green adhesive foam. The target always moved under constant acceleration, either accelerating (*a* = ±120 cm/s^2^) or moving with constant speed (constant motion, *a* = 0). The average speed of the target when in the striking zone on all trials was: 173.59 cm/s (173.65 ± 0.25, 173.65 ± 0.15, and 173.47 ± 0.23 cm/s for constant, accelerated and decelerated motion, respectively); 193.91 cm/s (194.12 ± 0.28, 194.15 ± 0.23, and 193.45 ± 0.26 cm/s); and 214.42 cm/s (214.48 ± 0.33, 214.57 ± 0.25, and 214.21 ± 0.21 cm/s). These values were estimated when the target’s centre reached the central position of the striking zone and the variability around the mean of these values is related to motor jitter.

The time for which the target was visible up to the moment of interception (viewing time) was varied randomly over trials within a range of 1.0–1.2 s (mean = 1.1 s). Viewing time was controlled through an electronically controlled liquid crystal display (LCD) goggles which could be switched between an opaque and a transparent state. The goggles were custom made using a normal pair of safety goggles (Monarch, model 061-clear), to which two layers (one internal and another external) of an LCD film were attached. The LCD film (Smart-Tint™, Reflex® Glass) changes its state from opaque (OFF mode) to transparent (ON mode) when activated with an electric current of 65 V (±5 V) AC and 1 AMP (OFF–ON response time of 100 ms). In opaque mode, the LCD film has an internal haze of 90% and a visible light transmission of 50%, and, respectively, 8 and 70% when in transparent mode. All participants wore these goggles during the whole experiment.

Infrared emitting diodes (IREDs) were fixed to the carriage in which the target was housed and to the base of the plastic block in which the handle was mounted. The positions of these IREDs were sampled at 200 Hz during experimental trials using an Optotrak Certus® (Northern Digital Inc.) optoelectronic movement recording system and stored on computer disc.

### Design and procedures

Before the experiment, participants received general instructions about the task and the equipment used in the experiment. They were instructed to grasp the handle of the sliding manipulandum and to strike the target using one single and continuous movement of their arm, avoiding moving a short distance, slowing down and speeding up, or stopping and then moving the remaining distance to the target. Whether or not the participants followed these instructions was noted by the experimenter in each trial during the data collection and data reduction (i.e. presence of minima in the speed profile), and later excluded from the data analysis (less than 2% of the total amount of trials were excluded). Participants were informed that the trial would start as soon as the LCD goggles became opaque and that after some time, the goggle would became clear allowing them to visualize the target approaching. The time between switching the goggles from opaque to clear at the beginning of the trial ranged from 3 to 3.5 s.

Participants were randomly allocated to one of the three experimental conditions according to the type of acceleration of the approaching targets: acceleration, deceleration, or constant speed. Participants in the acceleration condition intercepted targets moving with a constant acceleration (*a* = +120 cm/s^2^), whereas the targets moved with constant deceleration (*a* = − 120 cm/s^2^) in the deceleration condition. Targets in the constant speed condition did not accelerated and moved with constant speed (*a* = 0).

Participants performed a total of 147 trials equally distributed for each target speed (slowest, medium and fastest) and pseudorandomly presented, always avoiding identical speed conditions in consecutive trials. Speeds conditions were defined as a function of the speed of the target at the interception zone. We adopted this procedure to keep the temporal precision for each speed condition the same across the two experimental groups; regardless of the type of acceleration, the targets were moving at. Participants performed all the trials in two blocks of trials with a 3 min resting period just after the 72th trial. In each group, participants were exposed to different sequences of trials which were generated by the computer at the beginning of each session.

### Data reduction

All data reductions were performed using the custom LabVIEW™ software (version 8.1, National Instruments Inc.) using the standard data processing algorithms. Any missing data of the IREDs were interpolated by cubic spline, as long as the number of missing samples did not exceed 10% of the sampling frequency (200 Hz). The position data time series were digitally filtered by dual pass through a second-order Butterworth filter with a cut-off frequency of 20 Hz and then interpolated (using cubic spline) to 1000 Hz. This software re-sampling post-filtering is essentially equivalent to hardware sampling at 1000 HZ, since the original sampling (200 Hz) was well above the Nyquist limit for human limb movements—such movements do not contain signal frequencies much above 10 Hz (e.g., Winter 2009). After this procedure, the position data time series were numerically differentiated once and twice to derive the speed and acceleration data of the target and intercepting effector, respectively. Movement onsets were calculated from the tangential speed time series using the two-stage algorithm B suggested by Teasdale et al. (1993). The algorithm first determines the sample (S1) at which the time series first exceeds 10% of its maximum value. Then, working back from S1 it finds the first sample (S2) at which speed reaches 10% of the speed value at S1. Working forward from S2, the final step of the algorithm locates the onset being the sample at which speed equals the average value plus the standard deviation between S1 and S2. The time at which the target was hit as well as the temporal error were estimated from the position time series of the manipulandum IRED and the target IRED less a small offset to take into account the amount the bat IRED was displaced from the surface of the bat and the distance the target IRED was displaced relative to the target surface. The sample at which the Y-position of the manipulandum reached the Y-position occupied by the target determined the time of target strike when the target was actually hit.

### Dependent measures and statistical analysis

#### Temporal errors

The main measure in this experiment was the temporal error (TE), which is defined as the difference between the time when the tip of the bat reaches the Y-position of the target’s plane of motion and the time when the target’s centre reached the Z-position, where the tip of the bat crossed the Y-position of the target’s plane of motion. The mean and the standard deviation of the temporal error over a series of trials are the constant temporal error (CTE) and the variable temporal error (VTE), respectively. In this experiment, we also analysed the hit rate (HR), defined as the percentage of targets contacted with the tip of the bat. For each participant, the 147 trials were divided into 7 blocks of 21 trials and averaged to give estimates of HR and CTE for each condition. For the calculation of VTE, we computed the standard deviation of the same 21 trials. Because HR is based on the percent of hits and follows a binomial distribution, the arcsine squared root transformation was used to analyse this variable as recommended by Hogg et al. (2012).

#### Movement trajectories

For the analysis of movement trajectories, we analysed movement time (MT), defined as the time when the subject start moving the manipulandum, relative to the moment that the centre of the moving target reaches the Z-position, where the tip of the bat crosses the target’s plane of motion and target strike, and the percentage of mono-phasic and bi-phasic movements. In a previous study, movements with inflection points in the speed profile were observed when performing this interception task (Tresilian and Lonergan 2002). These inflection points appear as minima in the acceleration profiles. Movements of this type have been interpreted as compound movements made up of two or more component submovements (Milner 1992; Rohrer and Hogan 2003) with later submovements possibly being corrections to errors in the initial movement (e.g., Meyer et al. 1988). We examined whether such movements were present in the data recorded using the same procedure used by Tresilian and Plooy (2006).

Minima in the acceleration profile were used to provide an estimate of the number of components in a particular movement. A one-component movement (mono-phasic movement) is one with no minima in the acceleration profile, a two-component (bi-phasic) movement has one minimum, and so on. The criteria for defining an acceleration minimum were chosen, such that the selected minima were associated with visually identifiable inflection points in the speed profile. It was found that this was achieved if the depth of a minimum was greater than 2% of the maximum overall acceleration (Tresilian and Plooy 2006). Depth was defined as follows: any minimum has acceleration peaks to the left and right, and the difference between the value of the acceleration at the lowest of the two peaks and at the minimum point is the depth of the minimum. This criterion ensured that none of the small fluctuations, evident in some acceleration profiles, were included as minima.

#### Statistical analysis

Effects of experimental conditions on the above described variables were analysed through a three-way Mixed Design ANOVA (3 Groups × 3 Target Speeds × 7 Blocks of Trials). Departures from sphericity were verified through the Mauchley’s test, and when necessary, the Greenhouse–Geisser’s method was used to correct the degrees of freedom. Differences between main factors were further assessed using the Tukey’s post-hoc procedure when sphericity was verified and the Bonferroni procedure when it was not. All reported differences were significant at the *P* level of 0.05.

## Results

### Temporal errors

**Fig. 2:**
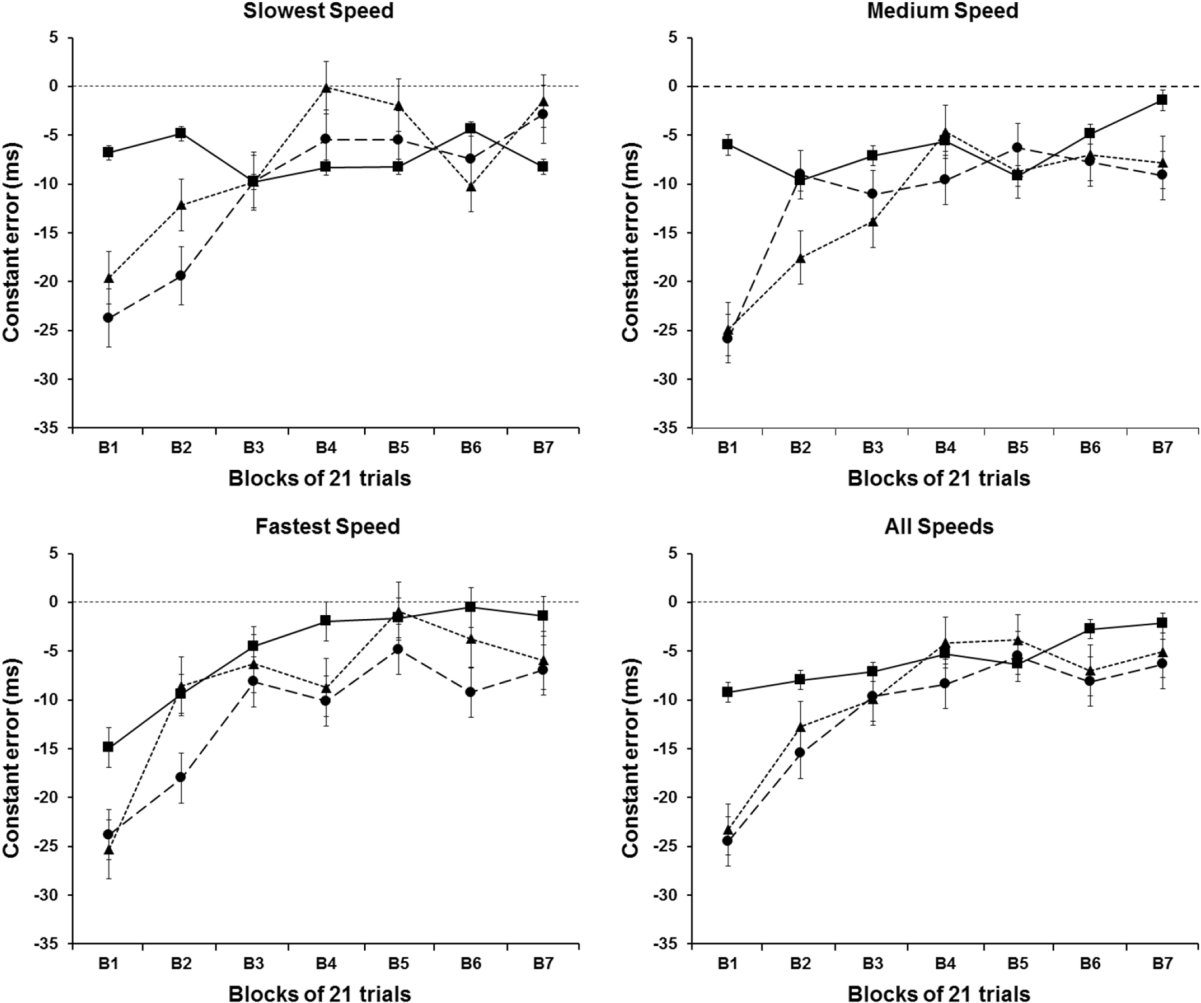
CTE means as a function of the blocks of trials. *Different symbols* represent different experimental conditions: (*triangle*) Acceleration group; (*filled circle*) Constant group; and (*filled square*) Deceleration group

Figure 2 shows the means of CTE for each of experimental condition in function of blocks of trials. Different graphs represent the results at each speeds condition, with the last graph plotting the average of these three speeds condition for each experimental condition. The results were virtually the same in each speed condition. Participants became more temporally accurate with practice, regardless of their experimental condition. Surprisingly, the results did not indicate any difference among the three experimental conditions. This pattern was confirmed by a statistically reliable effect on blocks factor [*F*_(2,661, 47,900)_ = 9.364, *P* < .001], and no reliable effect for groups and speeds factors, or any interactions among the three main factors (*P* > .05). Overall, the post-hoc analyses on the blocks factor revealed that the first two blocks of trials were different from the remaining blocks of trials (*P* < .05).

**Fig. 3:**
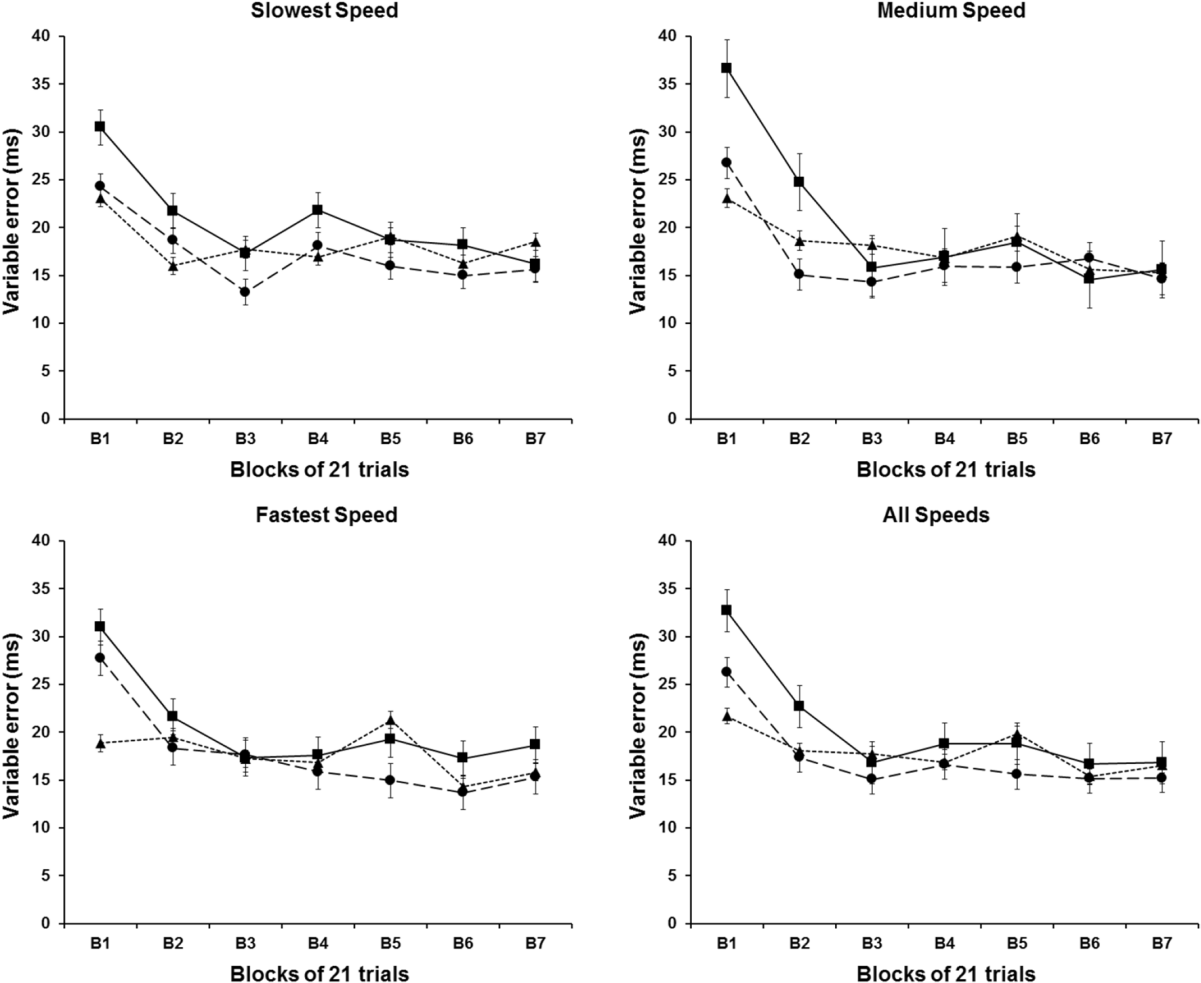
VTE means for each speed condition as a function of the blocks of trials. *Different symbols* represent different experimental conditions: (*filled triangle*) Acceleration group; (*filled circle*) Constant group; and (*filled square*) Deceleration group

Following the results of the CTE, the results for the VTE were very similar, as shown in Fig. 3. Likewise, the results for the CTE participants improved their performance with a decrease of the VTE in the first two blocks of trials, and again, no difference seems to exist between the three experimental conditions. The inferential analysis revealed a statistically reliable effect on the block factor [*F*_(2,615, 47,067)_ = 16.162, *P* < .001], and no reliable effect for groups and speeds factors, or any interactions among the three main factors (*P* > .05). Post-hoc analyses on the blocks factor revealed that participant’s performance was more variable on the first block of trials when compared to the remaining blocks of trials (*P* < .05).

**Fig. 4:**
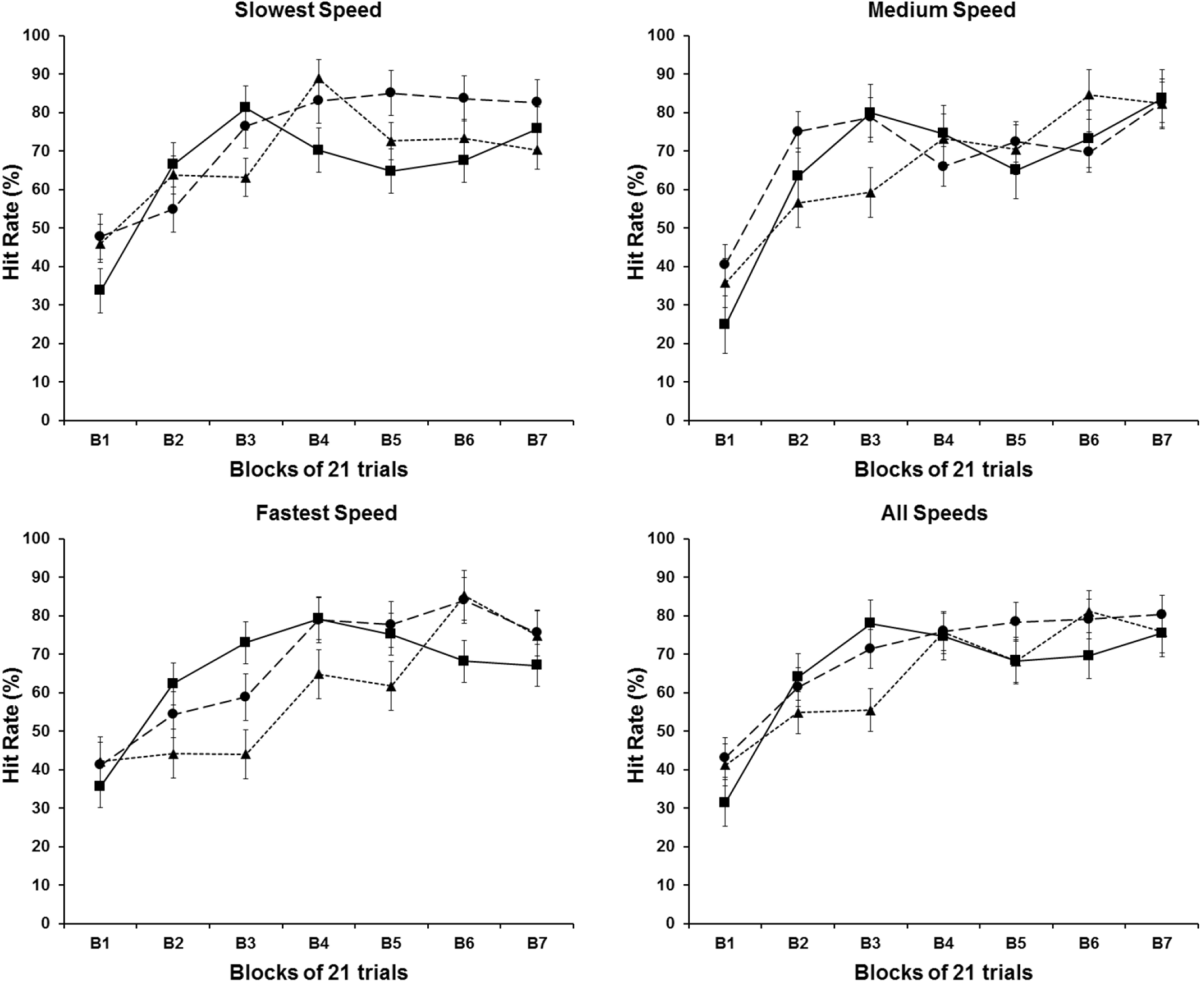
HR means for each speed condition as a function of the blocks of trials. *Different symbols* represent different experimental conditions: (*filled triangle*) Acceleration group; (*filled circle*) Constant group; and (*filled square*) Deceleration

Reflecting the results for the CTE and the VTE, the results of the HR show that participants improved their performance by hitting more targets as they practiced the task (Fig. 4). Participants started the practice missing more than half of the targets, but by the second block of trials, they were able to hit more targets than miss them. The three groups had a very similar performance in terms of hits and misses throughout the whole experiment. Once again, the inferential analysis revealed a statistically reliable effect on the block factor [*F*_(6, 108)_ = 21.772, *P* < .001], and no reliable effect for groups and speeds factors, or any interactions among the three main factors (*P* > .05). The post-hoc analyses on the blocks factor revealed that participants hit fewer targets on the first two blocks of trials when compared with the following blocks of trials (*P* < .05).

### Movement trajectory

**Fig. 5:**
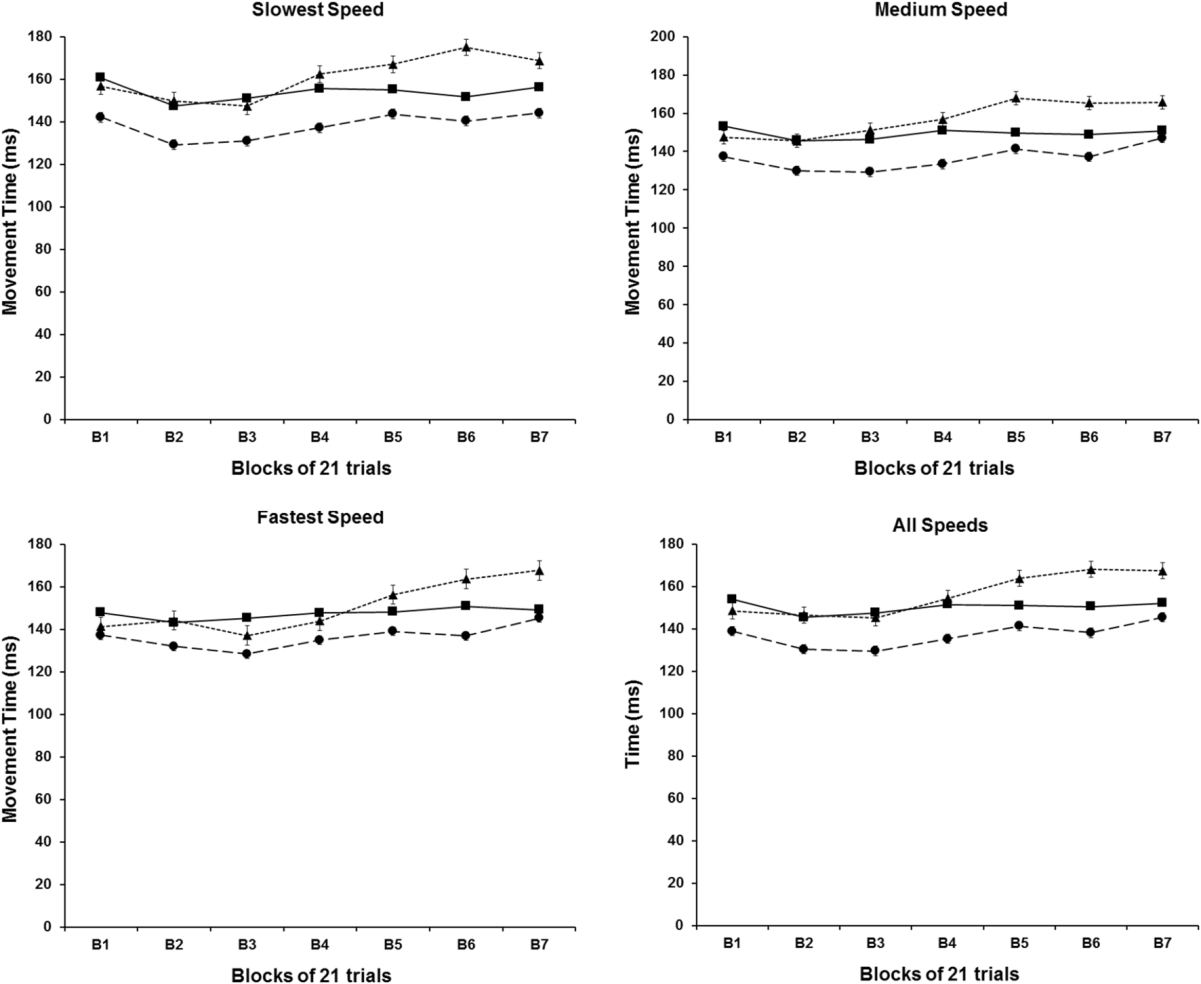
MT means for each speed condition as a function of the blocks of trials. *Different symbols* represent different experimental conditions: (*filled triangle*) Acceleration group; (*filled circle*) Constant group; and (*filled square*) Deceleration group

MT data for each of experimental condition in a function of blocks of trials is presented in Fig. 5. Different lines represent the mean of movement time for each experimental condition. The results for the MT show an overall pattern for the three experimental groups, with moment duration being similar among the group conditions and between 130 and 170 ms throughout the whole experiment. These results were confirmed by the three-way Mixed Design ANOVA, which did not detect any statistically reliable effect for groups, blocks and for the interaction among the three factors (*P* > .05), but detected a statistically reliable effect for the speeds factor [*F*_(1,642, 29,548)_ = 26.809, *P* < .001]. The post-hoc analyses on the speeds factor revealed that participants’ movements towards the fastest targets were briefer than towards the medium (*P* < .002) and slowest (*P* < .001) targets, and movements towards medium targets were briefer than towards the slowest targets (*P* < .001).

**Fig. 6:**
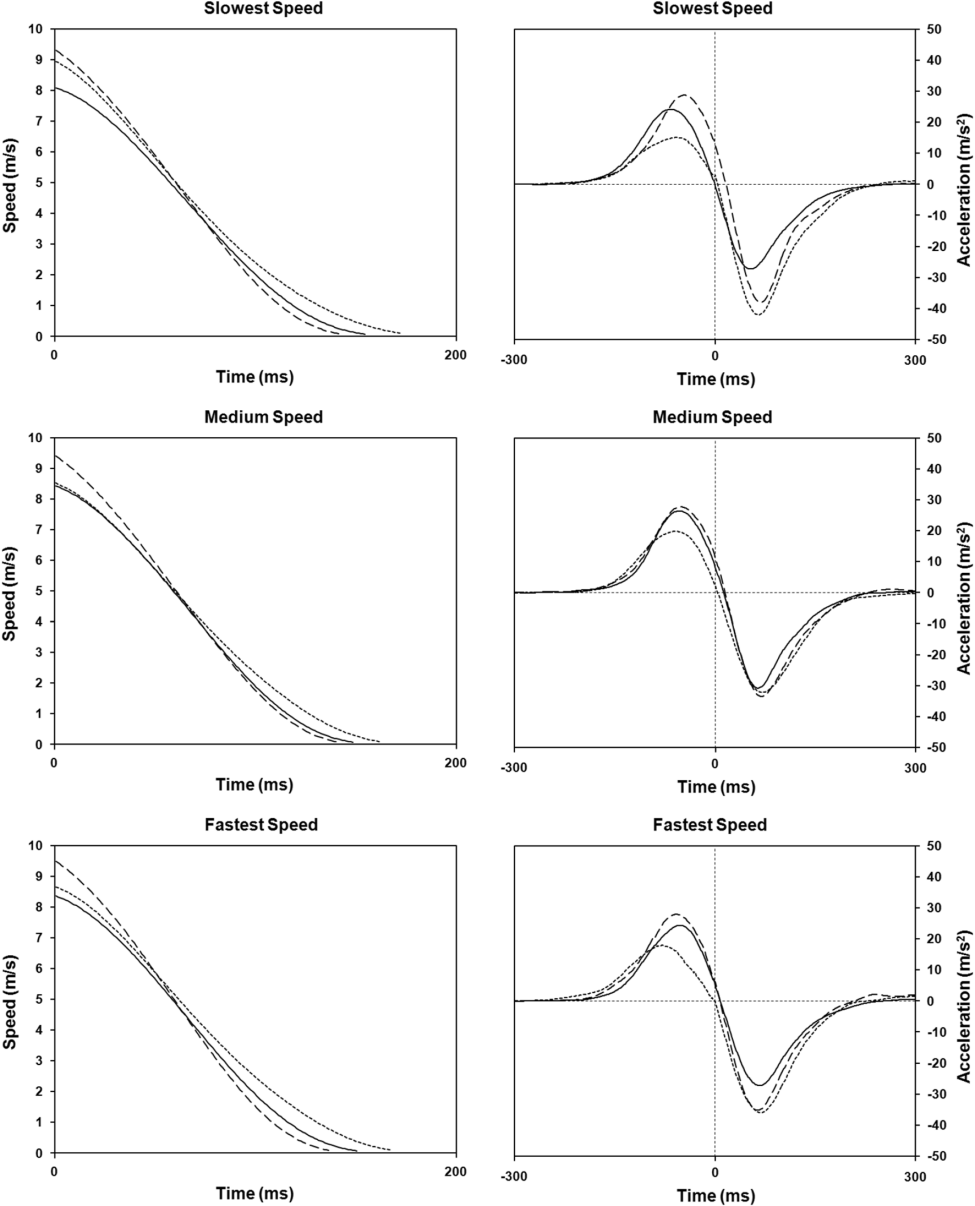
Speed and acceleration profiles (*left* and *right graphs*, respectively) plotted as a function of time. Data points were normalized to the average moment time. Movement speed and acceleration were aligned on interception time (time 0). Different lines represent different experimental conditions: (*dotted line*) Acceleration group; (*dashed line*) Constant group; and (*thin line*) Deceleration

Figure 6 shows the means of the speed and acceleration data for each of experimental condition as a function of time. Different lines of graphs represent the results at each speeds condition with the graphs on the left showing the speed profile and the graphs on the right showing the acceleration profile. The results showed that the zero-crossing of movement acceleration was very close to the interception time (time 0), indicating that subjects generated maximum momentum to hit the moving target. Concerning the type of movements, virtually 100% of all movements, regardless of the speed condition or the acceleration condition (i.e., groups), was mono-phasic in all participants. These results were confirmed by the three-way Mixed Design ANOVA, which did not detect any statistically reliable effect (*P* > .05).

## Discussion

A number of studies have investigated how people time the manual interception of accelerating targets and reported results consistent with the idea that acceleration (rate of change of speed) information is not used (e.g., Benguigui et al. 2003; Lee et al. 1983; Michaels et al. 2001; Port et al. 1997). A series of studies over the last two decades have demonstrated that people can, with practice, learn to utilize acceleration information to control the timing of interceptions by acquiring knowledge about regularities in acceleration over repeated trials and using that knowledge in the timing of subsequent attempts (Zago et al. 2009). It was not initially clear whether this ability to take acceleration into account was restricted to accelerations of gravitational magnitude (≈9.81 m/s^2^) and that other accelerations (which are almost always of substantially smaller magnitude) are ignored. However, recent evidence shows that there are situations in which smaller amplitude accelerations are not ignored (e.g., de Rugy et al. 2012; La Scaleia et al. 2014; Tresilian and Lonergan 2002). The results reported here to extend these latter results to show that people can learn to take smaller than gravitational magnitude accelerations (1.2 m/s^2^) into account for both positive (increasing speed) and negative (decreasing speed) accelerations with relatively little experience (<60 trials) and that this does not involve the use of cues regarding the source of the acceleration (an incline) or about the initial conditions of the motion.

The results of this experiment clearly showed that participant’s interceptive timing performance, assessed in terms of temporal errors and hit rates, improved systematically over the first three blocks of trials (63 trials) with the larger part of the improvements occurring within the first two blocks (42 trials). These improvements were not only observed for accelerating/decelerating targets, but also for constant velocity targets: all three groups of participants improved in a somewhat similar way and from similar starting points. In the later trials (from trial 64 on), changes in performance were much smaller in all dependent variables. The constant error data and hit rate data (Figs. 2, 4) showed some small changes in performance over blocks 4–7, but the variable errors remain essentially unchanged over these blocks in all groups. It is expected that performance improvements over initial trials would be observed when the target moved at constant speed, since the participants require a period of familiarization with the task to perform effectively as observed in the previous studies using a similar hitting task and constant speed targets (e.g., Tresilian and Plooy 2006). It was not expected that performance with accelerating and decelerating targets would be so similar to that observed with constant speed targets.

The hypothesis that accelerations are ignored and the hypothesis that a period of learning is needed to acquire an internal model of the acceleration would both predict that performance would initially be better for the constant speed target group than for the accelerating target groups. Exactly how much better depends upon the errors incurred by not taking accelerations into account. These errors can be estimated if we assume a control strategy in which the phase of a hitting movement completed prior to hitting the target (in effect, the acceleration phase of the movement, Fig. 6) is performed visually open loop if its duration (the movement time) is less than about 150 ms (which is consistent with available data, see Elliott et al. 2010; Tresilian 2005; Tyldesley and Whiting 1975). Consistent with this assumption, the analysis of movement trajectories did not reveal the presence of multi-phasic movements, which would indicate the presence of online corrective submovements (e.g., Meyer et al. 1988).

According to the tau-hypothesis, an interceptive movement is triggered when the perceived time to arrival of the moving target reached a criterion value after a short visuo-motor delay (Lee 1980; Lee and Reddish 1981). The lowest estimates for this delay are around 100 ms (Brenner et al. 1998; Marinovic et al. 2009) and so an appropriate value of perceived time to arrival to use to initiate the interception would be 150 + 100 ms. Thus, if it is assumed that the timing of movement is based on initiating a brief ballistic movement when perceived time to arrival reached a value of about 250 ms, the temporal error expected if acceleration were not taken into account can be estimated using Newton’s equations for uniformly accelerated motion. The expected errors for the three speeds (173.6, 193.9, and 214.4 cm/s) are, respectively , +76, +73, and +70 ms (late errors) for the accelerating targets and − 18, −17, and − 15 ms (early errors) for the decelerating targets. For comparison, if a 200 ms value of time to arrival were used (perhaps to correct an ongoing movement), the corresponding expected errors would be +16, +14, and +13 and − 12, −11, and − 10 ms. We would expect, therefore, the initial (first block) temporal constant errors of the accelerating target group to be later than those of the constant speed target group and for those of the decelerating target group to be earlier than those of the constant target group. There was no evidence for such a pattern in the constant error data (Fig. 2); indeed, in some instances, the pattern present was in the opposite direction to that predicted (e.g., the deceleration groups errors were, on average, more positive rather than more negative than the constant speed groups errors). Thus, there was no evidence in the constant error data to suggest that accelerations were being ignored, even in the initial 20 trials.

Given that there was an overall tendency for early (−ve) temporal error (Fig. 2), it would not necessarily be expected that late (+ve) errors would be observed in early attempts to hit the accelerating target. However, the similarity of the constant errors in the three groups and the complete absence of the expected pattern were unexpected and difficult to account for in terms of existing hypotheses concerning how interceptions are timed. One possibility is that in early trials, participants are trying out alternative strategies for timing interceptive movements sufficiently accurately to hit the target and that with more to learn when the target accelerates (i.e., the need to acquire knowledge about the acceleration that can be incorporated into control of movement initiation), the ‘trying out’ process produces a greater variation in movement timing and hence a greater variation in timing errors (such a highly variable ‘trying out’ phase is characteristic of the early stages in instrumental learning and skill acquisition, e.g., Schmidt and Lee 2011; Woodworth 1899). This greater variation could mask the expected error pattern described above. The variable temporal error data (Fig. 3) do not provide any clear support for this hypothesis. There is evidence to indicate that the initial (first and second block) VTEs were greater in the decelerating target group than in the constant speed group (consistent with the lowest hit rate for the deceleration group in the first trial block), but no evidence to suggest that it was greater in the accelerating target group (the mean VTE in the first block was always the smallest in this group). Thus, the absence of the expected pattern—indeed, the fact that the means follow a pattern opposite to that expected—is currently hard to satisfactorily explain.

Overall, the results reported here extend previous results to show that in the absence of information about the conditions of accelerated motion (slope, starting position) that could provide cues about the acceleration and/or the time of arrival at an interception location, participants can learn to time their interceptions of continuously accelerated moving targets (both positively and negatively accelerated targets) with an accuracy similar to that achieved when the targets move at constant speed. For the particular task used in this experiment, participants were able to intercept accelerating targets with a high degree of effectiveness (between 60 and 80% hit rate) after fewer than 60 practice trials. These findings are consistent with recent data showing that the acquisition of knowledge about target acceleration (perhaps in the form of an internal model) established to be used when intercepting targets falling under gravitational acceleration is also involved (at least in some form) for other, less familiar accelerations of smaller magnitude (de Rugy et al. 2012; La Scaleia et al. 2014). The data presented here and in other studies are not sufficient to be able to state what kind of knowledge of acceleration and/or its effects is acquired: at this stage, it is only possible to say that some kind of knowledge is acquired, but this is not necessarily an estimate of the magnitude of the acceleration. Exactly what information is acquired is likely to depend upon the conditions and demands of the interceptive task, as the performer need only acquires sufficient knowledge to be able to perform the task effectively in the conditions experienced.
